# Exploring multidrug-resistant *Klebsiella pneumoniae* antimicrobial resistance mechanisms through whole genome sequencing analysis

**DOI:** 10.1186/s12866-023-02974-y

**Published:** 2023-09-02

**Authors:** Jing Yang, Kai Zhang, Chen Ding, Song Wang, Weiwei Wu, Xiangqun Liu

**Affiliations:** 1 Xuzhou Medical University, Xuzhou, 221004 Jiangsu China; 2 Clinical Laboratory, The Affiliated Xuzhou Municipal Hospital of Xuzhou Medical University, No. 269, Daxue Road, Tongshan District, Xuzhou, 221002 Jiangsu China; 3 Xuzhou Central Hospital, Xuzhou, 221009 Jiangsu China; 4 Dinfectome Inc, Nanjing, 210000 Jiangsu China; 5 Department of Respiratory and Critical Care Medicine, The Affiliated Xuzhou Municipal Hospital of Xuzhou Medical University, No. 269, Daxue Road, Tongshan District, Xuzhou, 221002 Jiangsu China

**Keywords:** *Klebsiella pneumoniae*, Antimicrobial susceptibility testing, Next-generation sequencing, Conventional microbiological test, Carbapenem

## Abstract

**Background:**

Antibiotic-resistant *Klebsiella pneumoniae* has emerged as a critical public health threat worldwide. Understanding the antimicrobial resistance mechanisms of multidrug-resistant *K. pneumoniae* (MDR-Kp) and its prevalence in time and space would provide clinical significance for managing pathogen infection.

**Methods:**

Eighteen clinical MDR-Kp strains were analyzed by whole genome sequencing (WGS), and the antimicrobial resistance genes and associated resistance mechanisms were compared with results obtained from the conventional microbiological test (CMT). The sequence homology across strains in our study and those previously collected over time from a wide geographical region was assessed by phylogenetic analysis.

**Results:**

MDR-Kp strains were collected from eighteen patients who had received empirical treatment before strain collection, with sputum (83.3%, 15/18) being the primary source of clinical samples. The commonly received treatments include β-lactamase inhibitors (55.6%, 10/18) and carbapenems (50%, 9/18). Using CMT, we found that all 18 strains were resistant to aztreonam and ciprofloxacin, while 14 (77.8%) showed resistance to carbapenem. Polymyxin B and tigecycline were the only antibiotics to which MDR-Kp strains were sensitive. A total of 42 antimicrobial resistance mechanisms were identified by WGS, surpassing the 40 detected by the conventional method, with 25 mechanisms shared between the two techniques. Despite a 100% accuracy rate of WGS in detecting penicillin-resistant strains, the accuracy in detecting cephalosporin-resistant strains was only at 60%. Among all resistance genes identified by WGS, *Klebsiella pneumoniae carbapenemase-2* (*KPC-2*) was present in all 14 carbapenem-resistant strains. Phenotypic analysis indicated that sequence type (ST) 11 isolates were the primary cause of these MDR-Kp infections. Additionally, phylogenic clustering analysis, encompassing both the clinical and MDR-Kp strains previously reported in China, revealed four distinct subgroups. No significant difference was observed in the sequence homology between *K. pneumoniae* strains in our study and those previously collected in East China over time.

**Conclusion:**

The application of WGS in identifying potential antimicrobial-resistant genes of MDR-Kp has demonstrated promising clinical significance. Comprehensive genomic information revealed by WGS holds the promise of guiding treatment decisions, enabling surveillance, and serving as a crucial asset in understanding antibiotic resistance.

**Supplementary Information:**

The online version contains supplementary material available at 10.1186/s12866-023-02974-y.

## Introduction

*Klebsiella pneumoniae* is a gram-negative bacterium that belongs to the family Enterobacteriaceae, which includes the two well-known genera *Salmonella* and *Escherichia* [1]. The common manifestations of *K*. *pneumoniae* infections are pneumonia, urinary tract infections, bloodstream infections, and wound infections [2]. Though *K*. *pneumoniae* is classically considered hospital-acquired and infects immunocompromised hosts as an opportunistic pathogen, hypervirulent and multidrug-resistant (MDR) groups have emerged for the presence of a large accessory genome composed of plasmids and chromosomally encoded genes [1, 3, 4]. As *K*. *pneumoniae* strains pose a significant clinical health threat worldwide, understanding factors responsible for pathogenicity and resistance will be crucial for effectively treating infected patients.

The treatment of *K*. *pneumoniae* infections has been challenging, and the resistance mechanisms are an active area of investigation. Beta-lactams are commonly used in the treatment of *K*. *pneumoniae* infections; however, the production of β-lactamase that hydrolyzes oxyimino-cephalosporins leads to acquired resistance and significantly impacts treatment efficacy [5]. Additionally, while broad-spectrum antibiotics such as carbapenems and fluoroquinolones are typically the drug of choice to treat infections caused by extended-spectrum β-lactamase (ESBL)-producing *K*. *pneumoniae* [6], carbapenem-resistant *K*. *pneumoniae* (CR-Kp) has been linked to resistance against these drugs, resulting in an escalated global risk of disease [7]. Besides, MDR *K*. *pneumoniae* (MDR-Kp) may also arise after acquiring resistance to second-line antibiotics treatment against ESBL-producing and CR-Kp strains [8]. More recently, *K*. *pneumoniae* vaccination has emerged as a promising and innovative strategy to effectively defeat this life-threatening pathogen. Ranjbarian et al. conducted a systematic review of different vaccines (i.e., conjugate, protein, polysaccharide, lipopolysaccharide) using animal models from 35 studies [9]. Although these attempts have shown effectiveness, major challenges in vaccination therapy persist, including low vaccine responses in immunocompromised patients, time-consuming and costly processes, and safety concerns. Indeed, there is currently no approved and globally available *K*. *pneumoniae* vaccine. Kleb4V, a tetravalent bioconjugate vaccine, is the only unlicensed vaccine candidate in the phase 1/2 clinical trial (NCT04959344), which marks the first-time-in-human evaluation of the vaccine’s safety and immunogenicity against *K*. *pneumoniae* [10]. Hence, efforts should be made to optimize treatment decisions and find alternative antigens for *K*. *pneumoniae* vaccination, as well as apply new strategies to provoke robust immune responses in animal models.

According to the China Antimicrobial Surveillance Network (CHINET), the resistance rate of *K*. *pneumoniae* to carbapenem antimicrobial agents, such as imipenem and meropenem, increased by eight times from 2005 to 2021 (3.0%–23.8%) [11]. The isolation rate of MDR strains also experienced a dramatic increase from 0.3% to 3.5% from 2008 to 2016. Critical geographical differences were observed in *K*. *pneumoniae* resistance in China, with East China having higher isolation rates than others. Furthermore, it was worth noting that public health surveillance data for this important pathogen was scarce during the outbreak of COVID-19. Considering the clinical importance of *K. pneumoniae* infections and their high prevalence in East China, gaining insight into how these emerging MDR strains acquire resistance to antimicrobial treatments and how they vary from one another becomes imperative for better disease control.

In this study, we retrospectively analyzed 18 MDR-Kp strains that were isolated from two tertiary first-class hospitals in Xuzhou, a nine-million population city in East China between December 2021 and April 2022, using conventional microbiological test (CMT) and next-generation whole genome sequencing (WGS). The clonal relationships of *K*. *pneumoniae* strains were analyzed by MLST, and the genetic relatedness of strains was compared with those previously identified in the same geographical region to evaluate the epidemiology of *K*. *pneumoniae* infections.

## Materials and methods

### Patients and MDR-Kp strain isolation

This study was conducted in accordance with the declaration of Helsinki and approved by the Ethics Committee of the Affiliated Xuzhou Municipal Hospital of Xuzhou Medical University (No. xyyll[2021]116). All patients provided written consent to participate and publication. All patients were empirically treated before strain culture, and the efficacy of treatment was evaluated after 72 h. Primary considerations for empirical treatment were as follows: (i) the local bacterial epidemiology and the associated antimicrobial resistance; (ii) distinguishing between community- and hospital-acquired infections. Clinical samples, including sputum, urine, and blood, were collected from patients, followed by *Klebsiella pneumoniae* isolation for antimicrobial susceptibility testing (AST) using the VITEK-2 Compact system (bioMérieux, France).

### Whole genome sequencing

The genomic DNA of the isolated MDR-Kp strains was extracted using PureLink Genomic DNA Mini Kit (Invitrogen, United States) according to the manufacturer’s instructions. The quantity and quality of DNA were assessed by Qubit 2.0 (Thermo Fisher Scientific, United States) and NanoDrop 2000 (Thermo Fisher Scientific, United States), respectively. DNA libraries were prepared using the KAPA Hyper Prep kit (KAPA Biosystems, United States) and were paired-end sequenced on the Illumina NovaSeq platform using 150 bp fragments (Dinfectome Inc., China). Low-quality reads, adaptor contaminations, duplications, and short reads of less than 36 bp were removed as previously described [12]. The filtered sequencing reads were aligned to the reference *Klebsiella pneumoniae* subsp. pneumoniae HS11286 [13] using the Burrows-Wheeler Alignment (BWA, v0.7.5) tool [14]. Genotypes were called for all samples individually with HaplotypeCaller from the Genome Analysis Tool Kit (GATK) (v3.3.0) using the default settings [15]. To identify drug-resistant genes, we conducted a comparison of sequencing reads with the Comprehensive Antibiotic Resistance Database (CARD 2023, https://card.mcmaster.ca/) using ResFinder 2.1 [16]. CARD provides both the reference database and toolsets based on detailed controlled molecular sequences and mutation data of antibiotic resistance genes [17, 18]. Srst2 [19] was used to detect multilocus sequence typing (MLST) using *Klebsiella pneumoniae* MLST sequences and definitions obtained from the PubMLST (https://pubmlst.org/).

### Single nucleotide polymorphism (SNP) discovery and phylogenetic construction

The sequencing reads of the 87 common *K. pneumoniae* strains were downloaded from the Bacterial and Viral Bioinformatics Resource Center (BV-BRC, https://www.bv-brc.org/). The primary inclusion criteria for the strain’s genome sequence were as follows: (i) the assembly level was complete; (ii) genome contamination was less than 1%; (iii) genome completeness exceeded 99%; (iv) passed the NCBI taxonomy check; (v) strains were collected from various regions across China. We used the available *K. pneumoniae* whole genome(https://www.ncbi.nlm.nih.gov/genome/815) as a reference for mapping and identifying SNPs. Sequencing reads were aligned to the reference using BWA36 (v0.7.5). Default parameters were used, allowing up to four mismatches and one gap when aligning reads to the genome. Alignments were converted from sequence alignment map (SAM) format to sorted, indexed binary alignment map (BAM) files62 (SAMtools v0.1.18). The Picard tool (v1.103; http://broadinstitute.github.io/picard) was used to remove duplicate reads. Genotypes were called for all samples individually with HaplotypeCaller from the Genome Analysis Tool Kit (GATK)38 (v3.3.0) using the default settings. The variant calls were filtered using a minimum PHRED quality threshold of 20 and a minimum and maximum variant coverage of 10 and 500 reads, respectively. SNPs with a deletion rate greater than 80% were excluded. All filter SNPs of the 87 common strains and 18 clinical strains were used to reconstruct a phylogenetic tree by the RAxML program with a gamma distribution and a general time-reversible model under 500 bootstrap repeats. Principal component analysis was performed to compare regional differences among bacteria strains.

## Results

### Patient characteristics

Based on the CMT result, MDR-Kp strains were defined as those resistant to ≥ 3 antimicrobial classes in addition to ampicillin, to which all *K*. *pneumoniae* infections are intrinsically resistant. Accordingly, a total of 18 clinical isolates were identified as MDR-Kp and subsequently analyzed by WGS to identify drug-resistant genes. Most patients (72.2%, 13/18) were male and above 65 years of age (Table 1). Sixteen patients have been hospitalized for more than two weeks, and nine underwent treatments in the Intensive Care Unit (ICU) within 30 days before strain culture. The most common comorbidities in these patients were brain disease (94.4%, 17/18) and respiratory disease (94.4%, 17/18), followed by hypertension (55.6%, 10/18) and heart disease (50%, 9/18). Notably, all patients had received empirical treatment before the collection of clinical isolates. The empirical treatment strategy for patients primarily relied on the local bacterial epidemiology and infection types, particularly distinguishing between community- and hospital-acquired infections. For community-acquired infections, antimicrobial drugs that effectively target the most identified bacteria were administered, with a subsequent escalation to carbapenems if significant improvement was not observed. In the case of hospital-acquired infections in critically ill patients, where multiple risk factors for infection with drug-resistant bacteria existed, the empirical use of carbapenems was considered as means to rapidly control the infection and prevent further disease progression. Accordingly, 14 patients (77.8%) were treated with multiple antibiotics, nine patients (50.0%) received carbapenems, and five patients (27.8%) were administered corticosteroids and immunosuppressants as part of their treatment regimen. Besides, it was observed that patients had undergone invasive treatment procedures. The most prevalent type was urinary catheter (66.7%), followed by tracheal intubation (44.4%) and mechanical ventilation (38.9%).

**Table 1:** Clinical characteristics of patients (N = 18)

Characteristic	N (%)
Sex (Male)	13 (72.2)
Age (> 65 years)	13 (72.2)
Department	
Respiratory	5 (27.8)
Neurological	4 (22.2)
ICU	3 (16.7)
Neurosurgery	3 (16.7)
Chinese traditional medicine	1 (5.6)
Rehabilitation medicine	1 (5.6)
Rheumatic immunology	1 (5.6)
Specimen types	
Sputum	15 (83.3)
Urine	2 (11.1)
Blood	1 (5.6)
ICU admission within 30 days before strain culture	9 (50)
Blood transfusion within 30 days before strain culture	3 (16.7)
Hospitalized for more than 14 days	16 (88.9)
Underlying diseases	
Brain diseases	17 (94.4)
Respiratory diseases	17 (94.4)
Hypertension	10 (55.6)
Heart diseases	9 (50.0)
Diabetes	3 (16.7)
Digestive system diseases	3 (16.7)
Urinary system diseases	3 (16.7)
Cancer	2 (11.1)
Immune system diseases	1 (5.6)
Complicated with fungal infection	1 (5.6)
Treatment received before strain culture	
Multiple antibiotics	14 (77.8)
β-lactamase inhibitors	10 (55.6)
Carbapenems	9 (50.0)
3rd or 4th generation Cephalosporins	7 (38.9)
Corticosteroids and immunosuppressants	5 (27.8)
Quinolones	5 (27.8)
Tetracyclines	3 (16.7)
Polypeptides	3 (16.7)
Aminoglycosides	2 (11.1)
Invasive procedure within 90 days before strain culture	
Urinary catheter	12 (66.7)
Tracheal intubation	8 (44.4)
Mechanical ventilation	7 (38.9)
Tracheostomy	5 (27.8)
Arteriovenous catheterization	3 (16.7)

### Antimicrobial susceptibility of clinical MDR-Kp strains

The antimicrobial susceptibility of MDR-Kp strains was tested against 40 antibiotics by CMT (Figure S1A and Table S1-S3). Here, we observed a high resistance rate of clinical isolates to β-lactams. Of note, all 18 cultured strains were resistant to aztreonam and ciprofloxacin (Table 2 and Table S1). In great contrast, only one tested strain (10%) was resistant to the cephalosporin antibiotic ceftazidime/avibactam, and all tested strains remained sensitive to tigecycline and polymyxin B (Table 2). Collectively, antimicrobial susceptibility testing (AST) results suggest that tigecycline and polymyxin B might serve as promising treatment strategies for patients infected with *K. pneumoniae* who have been previously exposed to broad-spectrum antibiotics.

**Table 2:** Selective antimicrobial susceptibility result by CTM

Antibiotics	Cultured strains	Resistant strains	Resistance rate (%)
Aztreonam	18	18	100
Ciprofloxacin	18	18	100
Amoxicillin/Clavulanic acid	18	17	94.4
Levofloxacin	18	17	94.4
Cefepime	18	16	88.9
Piperacillin/Tazobactam	18	15	83.3
Imipenem	18	14	77.8
Tobramycin	18	13	72.2
Amikacin	18	12	66.7
Trimethoprim/Sulfamethoxazole	18	8	44.4
Ceftazidime/Avibactam	10	1	10
Tigecycline	9	0	0
Polymyxin B	10	0	0

### Deciphering the putative drug-resistance genes in MDR-Kp through whole genome sequencing

The optimal treatment option for MDR-Kp infections has not yet been well established. Here, we aimed to identify potential drug-resistance genes in clinical MDR-Kp isolates through WGS. By understanding the specific resistance mechanisms of MDR-Kp clinical isolates, clinicians can tailor the use of appropriate antibiotics, thereby effectively clearing the infection and improving patient clinical outcomes. Among the 42 potential resistance mechanisms detected through WGS, 25 (43.9%) were found to be associated with known drug-resistance mechanisms, which can be readily evaluated by CMT (Figure S1B; Table S4 and S5). Notably, *Klebsiella pneumoniae carbapenemase-2* (*KPC-2*) and *parC* had the highest detection rate (77.8%, 14/18), followed by *rmtB* and *sul2* (66.7%, 12/18) (Table 3). Next, we assessed the performance of WGS for detecting known mechanisms conferring resistance in MDR-Kp strains. For β-lactams, while WGS achieved a 100% sensitivity and accuracy in identifying penicillin-resistant strains, the performance dropped by 40% in predicting the sensitivity of *K. pneumoniae* to cephalothin and cefuroxime (Table 4). Regarding other frequently employed antibiotics, WGS demonstrated a moderate performance, exhibiting an accuracy ranging from 77.8 to 100%.

**Table 3:** List of drug-resistant genes identified by whole genome sequencing

Gene	Underlying drug resistance	No. strains	Detection rate (%)	Reference
KPC-2	Monobactam, carbapenem, cephalosporin and penam antibiotics	14	77.8	[29]
parC	Fluoroquinolones	14	77.8	[30]
rmtB	Aminoglycosides	12	66.7	[31]
sul2	Sulfonamides, i.e., sulfamethizole	12	66.7	[32]
SHV-134	Carbapenem, cephalosporin and penam antibiotics	11	61.1	[33]
tet(A)	Tigecycline and tetracycline	11	61.1	[34]
CTX-M-65	Cephalosporins	9	50.0	[34]
LAP-2	Fluoroquinolone, aminoglycoside, tetracycline and rifamycin antibiotics	9	50.0	[35]
AAC(3)-IId	Aminoglycosides	7	38.9	[36]
SHV-11	Carbapenem, cephalosporin and penam antibiotics	7	38.9	[37]
APH(6)-Id	Aminoglycosides, i.e., streptomycin	5	27.8	[38]
APH(3’’)-Ib	Aminoglycosides, i.e., streptomycin	4	22.2	[38]
dfrA14	Diaminopyrimidines, i.e., trimethoprim	4	22.2	[39]
sul1	Sulfonamides, i.e., sulfamethoxadiazole	3	16.7	[40]
TEM-1	Monobactam, cephalosporin, penam and penicillin antibiotics	3	16.7	[41]
AAC(6’)-Ib-cr	Aminoglycosides, i.e., ciprofloxacin and netilmicin	2	11.1	[42]
dfrA27	Diaminopyrimidines, i.e., trimethoprim	2	11.1	[43]
floR	Chloramphenicol, florfenicol and other chloramphenicol antibiotics	2	11.1	[44]
TEM-2	Monobactam, cephalosporin, penam and penicillin antibiotics	2	11.1	[45]
AAC(6’)-Ib9	Aminoglycosides, i.e., neomycin and gentamicin B	1	5.6	[46]
aadA	Aminoglycosides, i.e., spectinomycin and streptomycin	1	5.6	[47]
aadA2	Aminoglycosides, i.e., spectinomycin and streptomycin	1	5.6	[48]
CTX-M-15	Cephalosporins, i.e., ceftazidime and ceftriaxone	1	5.6	[49]
CTX-M-3	Cephalosporins, i.e., ceftazidime and ceftriaxone	1	5.6	[50]
gyrA	Fluoroquinolone, i.e., nalidixic acid	1	5.6	[51]
mel	Erythromycin, telithromycin, macrolide, lincosamide, streptomycin, tetracycline, oxazolidinone, chloramphenicol and pleuromutilin antibiotics	1	5.6	[52]
mphA	Macrolides	1	5.6	[53]
NDM-1	Carbapenem, cephalosporin, cephalosporin and penam antibiotics	1	5.6	[54]
OXA-1	Cephalosporins and penams	1	5.6	[55]
OXA-485	Cephalosporins and penams	1	5.6	[56]
QnrB6	Fluoroquinolone antibiotics, i.e., ciprofloxacin and levofloxacin	1	5.6	[57]

**Table 4:** WGS performance in identifying drug-resistance mechanisms of MDR-Kp strains

Type of antibiotics		Antibiotics	Sensitivity (%)	Specificity (%)	PPV (%)	NPV (%)	Accuracy (%)
β-lactams	Penicillins	Ticarcillin	100	/	100	/	100
		Piperacillin	100	/	100	/	100
		Amoxicillin/ Clavulanic acid	100	/	100	/	100
	Cephalosporins	Cephalothin	60	/	100	0	60
		Cefuroxime	60	/	100	0	60
		Cefotaxime	100	/	100	/	100
		Ceftazidime	100	/	100	/	100
		Cefepime	100	50	94.1	/	94.4
		Cefazolin	87.5	/	100	0	87.5
		Cefoxitin	57.1	100	100	25	62.5
		Ceftriaxone	100	/	100	/	100
	Carbapenems	Imipenem	87.5	100	100	50	88.9
		Meropenem	100	/	100	/	100
		Ertapenem	\	100	\	100	100
	Penicillin/β-lactamase inhibitor	Piperacillin/ Tazobactam	93.3	100	100	75	94.4
	Monobactams	Aztreonam	100	0	77.8	0	77.8
Quinolones		Moxifloxacin	90	/	100	/	90
		Norfloxacin	90	/	100	/	90
		Ciprofloxacin	83.3	/	100	0	83.3
		Levofloxacin	77.8	/	100	0	77.8
Aminoglycosides		Tobramycin	100	66.7	93.8	100	94.4
		Amikacin	100	100	100	100	100
Tetracyclines		Tetracycline	71.4	/	100	60	80
		Doxycycline	71.4	100	100	60	80

Value not available due to no sensitive strains (/) or no resistant strains (\) determined by conventional drug sensitivity test. PPV, positive prediction value; NPV, negative prediction value

### Exploring the genetic divergence of MDR-Kp strains in temporal and spatial scales

The diversity and complexity of MDR-Kp strains in our study were examined using the multilocus sequence typing (MLST) test [20]. The MLST is a nucleotide sequence-based approach developed for *K. pneumoniae* to characterize the genetic relationships among isolates. Four sequence types (ST) were identified, with ST11 being the predominant type (Table S6). This finding was consistent with others, suggesting that ST11 is the dominant cause of *K. pneumoniae* infections in China.

Next, we performed a single nucleotide polymorphism (SNP)-based phylogenic analysis to compare the genetic similarities and evolutionary relationships among the 18 MDR-Kp isolates in our study and 87 strains previously identified in China. As a result, MDR-Kp strains investigated in our study were primarily classified into four clusters. Notably, half of the strains were found clustered together with Hunan and Shanghai strains in clusters A, while six strains (33.3%) formed a distinct grouping in cluster B (Figure S2A). However, our analysis did not show any significant spatial or temporal differences in the distribution pattern of these strains (Figure S2B, C). This finding suggests a widespread prevalence of MDR-Kp strains in China, indicating a significant public health threat that necessitates immediate attention.

## Discussion

In this study, we utilized WGS to identify potential resistance mechanisms of MDR-Kp strains and compared that with CMT results. The sequence homology of *K*. *pneumoniae* strains isolated in local hospitals was also compared with those previously collected in a wider geographical region over time to uncover the diversity and complexity of this pathogen.

*K*. *pneumoniae* is one of the most common nosocomial pathogens in the world [21]. It is also a leading cause of ventilator-associated pneumonia among patients in the ICU [22, 23]. Consistently, we found that half of the patients had previous records of being treated in the ICU within 30 days before strain culture, including 2 and 4 patients who were later transferred from the ICU to the respiratory department and the neurology department, respectively (Table 1). This finding suggests that the ICU is the primary source of MDR-Kp infections in the two local hospitals, and the clinical use of antibiotics in the ICU should be more standardized. In addition, we found that 12 out of 18 patients (66.7%) were invaded by urinary tract-associated catheters within three months before strain culture. This finding supports the idea that the urinary tract is a common site of *K*. *pneumoniae* infection, with catheter-associated procedures being the primary cause of infection, possibly due to the ability of *K*. *pneumoniae* to form biofilms and adhere to catheters. Besides, we report that the primary underlying diseases associated with MDR-Kp infection were respiratory and brain diseases (17/18, 94.4%). However, given that *K. pneumoniae* is also the second leading cause of bloodstream infections, MDR-Kp strains may have originated from secondary infections that trace back to a known source.

MDR-Kp infections are typically associated with a high mortality rate; however, there is currently no standardized treatment for effectively controlling such diseases. Here, we aim to elucidate the mechanisms underlying antibiotic resistance in clinical MDR-Kp isolates within local hospitals using both conventional methods and WGS. Aztreonam is a vital treatment scheme for severe infection caused by *Enterobacter* species producing metallo-β-lactamases [24]. The CMT results in our study revealed that all MDR-Kp strains were resistant to aztreonam and ciprofloxacin, suggesting that these two antibiotics are likely ineffective against *K*. *pneumoniae* in the Xuzhou region. In addition, we noticed that the resistance rate of *K*. *pneumoniae* to ciprofloxacin was significantly higher than previously reported by CHINET (100% vs. 33%). We reasoned that this could be due to regional differences and limited sample size. Moreover, 77.8% of strains were identified to be CR-Kp. Since half of the patients had been previously treated with carbapenem antibiotics, we reasoned that *K*. *pneumoniae* might be susceptible to carbapenem resistance. Therefore, clinicians should be more cautious when prescribing such antibiotics. Lastly, we found that all examined MDR-Kp strains were sensitive to tigecycline and polymyxin B. Indeed, there have been previous reports showing that tigecycline effectively treats complex abdominal infections and community-acquired pneumonia caused by refractory bacterial infections [25]. Combining polymyxin B in antimicrobial treatment may provide better efficacy as it reduces the antibiotic dosage and eliminates the generation of drug-resistant strains [26]. Despite this promising result, given that polymyxin B is considered the last resort for treating CR-Kp infections, it is of clinical importance to develop other molecular/genetic tools to identify potential resistance mechanisms of the specific strain for optimal treatment decision-making and avoid misconduct of therapies.

In this regard, WGS is an unbiased approach that enables the broad identification of known and unexpected pathogens and provides auxiliary genomic information to predict drug resistance [27, 28]. In this present study, all WGS results were returned within 48 h, much shorter than the turnaround time of using traditional microbiological tests. Notably, we identified 42 potential resistance mechanisms of locally isolated clinical MDR-Kp strains using WGS, with *KPC-2* and *parC* being the most commonly identified drug-resistant genes among all these strains. While 25 resistance mechanisms were identified by both techniques, 15 were exclusively identified by CMT but not by WGS. Additionally, WGS showed false negative results in 11 strains, most of which were cephalosporin-resistant (5 strains) or quinolone-resistant strains (4 strains). Indeed, several factors may affect the number of mechanisms identified by both techniques, including the scope of CMT (available antibiotics), the reference database used against WGS data, and even treatment strategies implemented for patients before the collection of clinical samples. Therefore, these results comparing the identified resistance mechanisms between CMT and WGS need to be interpreted with caution. Nonetheless, the NGS-facilitated resistant gene detection holds great promise in supplementing the conventional testing approach for prompt treatment decision-making and clearance of pathogen infection.

A deeper understanding of *K*. *pneumoniae* diversity will be important for interpreting public health surveillance data and the implementation of novel control strategies against this pathogen. Here, we compared MDR-Kp strains to those previously identified in other regions of China and Xuzhou strains isolated between 2016 and 2021. MDR-Kp strains in this study were mainly ST11 and clustered in four categories. No significant spatial or temporal differences were observed, suggesting a wide geographical spread of clinically relevant MDR-Kp strains in East China. Therefore, close surveillance should be carried out in local hospitals to prevent the outbreak of MDR-Kp strains.

Despite these promising results, there are limitations to this study. First, our study has a relatively small sample size. Expanding the sample size is warranted to provide a more comprehensive view of the potential resistance mechanism of *K*. *pneumoniae* to antibiotics. Second, although WGS identified a broad range of antibiotic-resistant genes and promising resistance mechanisms, whether these drug-resistant genes are expressed *in vivo* awaits to be tested. Phenotypic tests might be considered to validate whether the underlying drug-resistant gene expresses the corresponding enzyme.

## Conclusion

To summarize, WGS demonstrated significant clinical implications to identify potential antimicrobial-resistant genes in MDR-Kp strains, which enables prompt treatment strategy adjustment and a better understanding of the resistance mechanism in MDR-Kp strains.

### Electronic supplementary material

Below is the link to the electronic supplementary material.

Supplementary Material 1

## Data Availability

All sequence reads were deposited into the NCBI Sequence Read Archive (SRA) database under the accession number PRJNA956822. All the other relevant data of the study are available from the corresponding authors upon reasonable request.

## Declarations

### Abbreviations

MDR, multidrug-resistant; MDR-Kp, multidrug-resistant *Klebsiella pneumoniae*; ESBL, extended-spectrum β-lactamase; CR-Kp, carbapenem-resistant *K*. *pneumoniae*; WGS, whole genome sequencing; ICU, intensive care units; MLST, multilocus sequence typing; ST, sequence type.
