# Supplementary material for: Exploring multidrug-resistant Klebsiella pneumoniae antimicrobial resistance mechanisms through whole genome sequencing analysis
Source: BMC Microbiol. 2023 Sep 2;23:245. doi: 10.1186/s12866-023-02974-y (PMC10474722; doi:10.1186/s12866-023-02974-y)
Supplement: Supplementary file 1 — Supplementary Material 1 [file 12866_2023_2974_MOESM1_ESM.docx]

**Supplementary Materials**

## Supplementary Figures


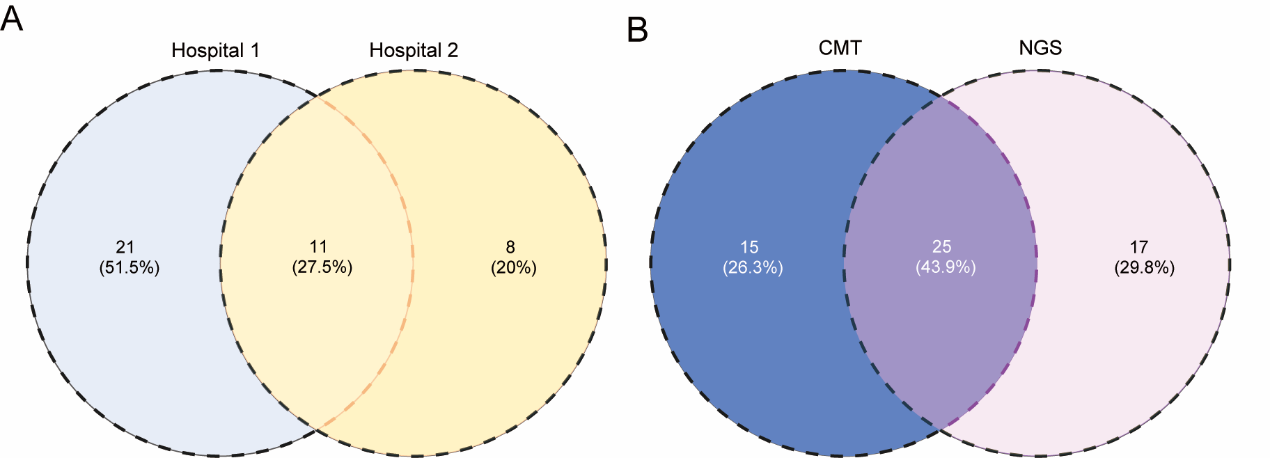


### Figure S1. Antimicrobial susceptibility tests

(A) Venn diagram showing the number and percentage of shared and unique antibiotics tested by conventional microbiology test (CMT) between the two hospitals. (B) Venn diagram showing shared and unique resistant mechanisms of MDR-Kp identified by CMT (n=40) and NGS (n=42).


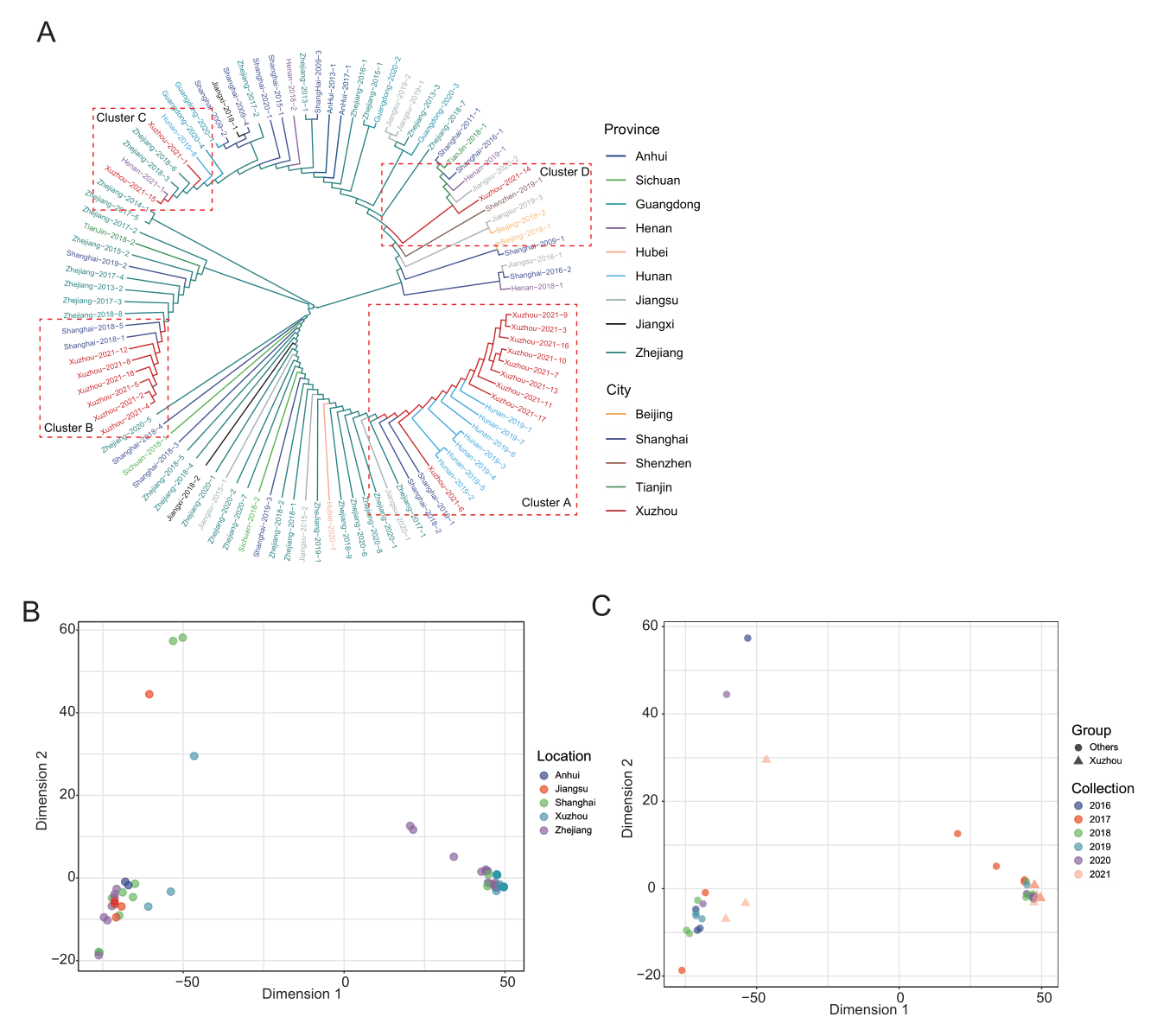


### Figure S2. Comparing MDR-Kp strains at spatial and temporal scales

(A) Phylogenic comparison of MDR-Kp strains in our study with those previously identified in China. Strains were color-coded based on their respective source regions. (B) Principal component analysis of the 18 MDR-Kp strains obtained from Xuzhou and an additional 57 strains collected from various locations in eastern China. (C) Principal component analysis of the Xuzhou strains and 26 additional strains collected from various locations in eastern China over the period from 2016 to 2021.

| **Table S1. Antimicrobial susceptibility testing results against shared antibiotics (n=11) in two research facilities** | | | | | | | | | | | | |
| --- | --- | --- | --- | --- | --- | --- | --- | --- | --- | --- | --- | --- |
| Hospital | Patient | Piperacillin/ Tazobactam | Amoxicillin/ Avulanic acid | Cefepime | Aztreonam | Imipenem | Tobramycin | Ciprofloxacin | Levofloxacin | Trimethoprim/ Sulfamethoxazole | Amikacin | Tigecycline |
|  | | MIC = µg/mL | | | | | | | | | | |
| H1 | P1 | ≥128 | ≥32 | ≥32 | ≥64 | ≥16 | ≥16 | ≥4 | ≥8 | 80 | ≥64 | 2 (S) |
| H1 | P2 | ≥128 | ≥32 | ≥32 | ≥64 | ≥16 | 8 | ≥4 | ≥8 | 80 | ≤2 (S) | 2 (S) |
| H1 | P3 | ≥128 | ≥32 | ≥32 | ≥64 | ≥16 | ≥16 | ≥4 | ≥8 | ≥320 | ≥64 | / |
| H1 | P4 | ≥128 | ≥32 | ≥32 | ≥64 | ≥16 | ≥16 | ≥4 | ≥8 | ≤20 (S) | ≥64 | / |
| H1 | P5 | ≥128 | ≥32 | ≥32 | ≥64 | ≥16 | ≥16 | ≥4 | ≥8 | ≤20 (S) | ≥64 | 1 (S) |
| H1 | P6 | ≥128 | ≥32 | ≥32 | ≥64 | ≥16 | ≥16 | ≥4 | ≥8 | ≥320 | ≥64 | / |
| H1 | P7 | ≥128 | ≥32 | ≥32 | ≥64 | ≥16 | ≤1 (S) | ≥4 | ≥8 | ≤20 (S) | ≤2 (S) | / |
| H1 | P8 | ≥128 | ≥32 | ≥32 | ≥64 | ≥16 | ＞16 | ≥4 | ≥8 | ≤20 (S) | 4 (S) | / |
| H1 | P9 | ≥128 | ≥32 | ≥32 | ≥64 | ≥16 | ＞16 | ≥4 | ≥8 | ≤20 (S) | ≥64 | 1 (S) |
| H1 | P10 | ≥128 | ≥32 | ≥32 | ≥64 | ≥16 | ＞16 | ≥4 | ≥8 | ≤20 (S) | ≥64 | 1 (S) |
| H2 | P11 | ≥128 | ≥32 | ≥64 | ≥64 | ≥16 | ≥16 | ≥4 | 2 | ≥320 | ≥64 | ≤0.5 (S) |
| H2 | P12 | ≥128 | ≥32 | ≥64 | ≥64 | 2 | ≥16 | ≥4 | ≥8 | ≤20 (S) | ≥64 | 2 (S) |
| H2 | P13 | ≥128 | ≥32 | ≥64 | ≥64 | ≥16 | ≤1 (S) | ≥4 | ≥8 | ≤20 (S) | ≤2 (S) | / |
| H2 | P14 | ≤4 (S) | ≥32 | 2 (S) | 16 | ≤1 (S) | 8 | 2 | 1 | ≥320 | ≤2 (S) | 2 (S) |
| H2 | P15 | 8 (S) | 16 | ≤1 (S) | ≥64 | ≤1 (S) | 4 (S) | ≥4 | ≥8 | ≥320 | ≤2 (S) | / |
| H2 | P16 | ≥128 | ≥32 | ≥64 | ≥64 | ≥16 | ≥16 | ≥4 | ≥8 | ≥320 | ≥64 | / |
| H2 | P17 | ≥128 | ≥32 | ≥64 | ≥64 | ≥16 | ≥16 | ≥4 | ≥8 | ≤20 (S) | ≥64 | 2 (S) |
| H2 | P18 | 8 (S) | ≥32 | ≥64 | ≥64 | 2 | ≥16 | ≥4 | ≥8 | ≤20 (S) | ≥64 | / |
| Abbreviations: MIC, minimal inhibitory concentration; R, resistant; S, sensitive | | | | | | | | | | | | |

**Supplementary Tables**

| **Table S2. Antimicrobial susceptibility testing results against unique antibiotics (n=21) tested in hospital 1** | | | | | | | | | | | |
| --- | --- | --- | --- | --- | --- | --- | --- | --- | --- | --- | --- |
| Patient | Ceftazidime/ Avibactam | Ticarcillin | Piperacillin | Cephalothin | Roxithromycin | Cefuroxime | Cefotetan | Cefpodoxime | Cefotaxime | Doxycycline | Minocycline |
|  | MIC = µg/mL | | | | | | | | | | |
| P1 | S | ≥128 | ≥128 | ≥64 | ≥64 | ≥64 | ≥64 | ≥8 | ≥64 | ≥16 | ≥16 |
| P2 | S | ≥128 | ≥128 | ≥64 | ≥64 | ≥64 | 32 | ≥8 | ≥64 | 8 | 8 |
| P3 | S | ≥128 | ≥128 | ≥64 | ≥64 | ≥64 | 32 | ≥8 | ≥64 | ≥16 | ≥16 |
| P4 | S | ≥128 | ≥128 | ≥64 | ≥64 | ≥64 | ≥64 | ≥8 | ≥64 | 8 | ≥16 |
| P5 | S | ≥128 | ≥128 | ≥64 | ≥64 | ≥64 | ≥64 | ≥8 | ≥64 | 1 (S) | ≤1 (S) |
| P6 | R | ≥128 | ≥128 | ≥64 | ≥64 | ≥64 | ≥64 | ≥8 | ≥64 | ≥16 | ≥16 |
| P7 | S | ≥128 | ≥128 | ≥64 | ≥64 | ≥64 | ≥64 | ≥8 | ≥64 | ≥16 | ≥16 |
| P8 | S | ≥128 | ≥128 | ≥64 | ≥64 | ≥64 | ≥64 | ≥8 | ≥64 | ≥16 | ≥16 |
| P9 | S | ≥128 | ≥128 | ≥64 | ≥64 | ≥64 | ≥64 | ≥8 | ≥64 | 1 (S) | 2 (S) |
| P10 | S | ≥128 | ≥128 | ≥64 | ≥64 | ≥64 | ≥64 | ≥8 | ≥64 | 2 (S) | 4 (S) |
|  | Ceftizoxime | Doripenem | Nalidixic acid | Moxifloxacin | Norfloxacin | Tetracycline | Ceftazidime | Cefoperazone/ Sulbactam | Meropenem | Polymyxin B |  |
| P1 | 32 | ≥8 | ≥32 | ≥8 | ≥16 | ≥16 | ≥64 | ≥64 | ≥16 | ≤0.5 (S) |  |
| P2 | 16 | ≥8 | ≥32 | ≥8 | ≥16 | 8 | ≥64 | ≥64 | ≥16 | ≤0.5 (S) |  |
| P3 | ≥64 | ≥8 | ≥32 | ≥8 | ≥16 | ≥16 | ≥64 | ≥64 | ≥16 | ≤0.5 (S) |  |
| P4 | 16 | ≥8 | ≥32 | ≥8 | ≥16 | 8 | ≥64 | ≥64 | ≥16 | ≤0.5 (S) |  |
| P5 | 16 | ≥8 | ≥32 | ≥8 | ≥16 | 4 (S) | ≥64 | ≥64 | ≥16 | ≤0.5 (S) |  |
| P6 | ≥64 | ≥8 | ≥32 | ≥8 | ≥16 | ≥16 | ≥64 | ≥64 | ≥16 | ≤0.5 (S) |  |
| P7 | 16 | ≥8 | ≥32 | ≥8 | ≥16 | ≥16 | ≥64 | ≥64 | ≥16 | ≤0.5 (S) |  |
| P8 | 32 | ≥8 | ≥32 | ≥8 | ≥16 | ≥16 | ≥64 | ≥64 | ≥16 | ≤0.5 (S) |  |
| P9 | 32 | ≥8 | ≥32 | ≥8 | ≥16 | 4 (S) | ≥64 | ≥64 | ≥16 | ≤0.5 (S) |  |
| P10 | 16 | ≥8 | ≥32 | ≥8 | ≥16 | 4 (S) | ≥64 | ≥64 | ≥16 | ≤0.5 (S) |  |
| Abbreviations: MIC, minimal inhibitory concentration; R, resistant; S, sensitive | | | | | | | | | | | |

| **Table S3. Antimicrobial susceptibility testing results against unique antibiotics (n=8) tested in hospital 2** | | | | | | | | |
| --- | --- | --- | --- | --- | --- | --- | --- | --- |
| Patient | Extended-spectrum beta-lactamase | Ampicillin | Cefazolin | Cefoxitin | Ceftriaxone | Gentamicin | Nitrofurantoin | Ertapenem |
|  | MIC = µg/mL | | | | | | | |
| P11 | Negative | ≥32 | ≥64 | ≥64 | ≥64 | ≥16 | 128 | / |
| P12 | Positive | ≥32 | ≥64 | ≥64 | ≥64 | ≥16 | ≥512 | / |
| P13 | Negative | ≥32 | ≥64 | ≥64 | ≥64 | ≤1 (S) | ≥512 | / |
| P14 | Positive | ≥32 | ≥64 | ≤4 (S) | ≥64 | ≥16 | 64 | / |
| P15 | Positive | ≥32 | ≥64 | 16 | 8 | ≤1 (S) | 64 | ≤0.5 (S) |
| P16 | Negative | ≥32 | ≥64 | ≥64 | ≥64 | ≥16 | ≥512 | / |
| P17 | Negative | ≥32 | ≥64 | ≥64 | ≥64 | ≥16 | 256 | / |
| P18 | Positive | ≥32 | ≥64 | ≥64 | ≥64 | ≥16 | ≥512 | / |
| Abbreviations: MIC, minimal inhibitory concentration; R, resistant; S, sensitive | | | | | | | | |

| **Table S4. Quality control data of filtered sequencing reads** | | | | | | | | | |
| --- | --- | --- | --- | --- | --- | --- | --- | --- | --- |
| Sample | Total reads | Total bases (M) | Q20 | Q30 | GC content | Mean depth | 1X coverage | 30X coverage | Fold80 |
| KA221B0389-XXXXH11RNF1-KX90 | 5454908 | 586.82 | 0.979 | 0.945 | 0.562 | 86 | 91 | 91 | 1.247 |
| KA221B0391-XXXXH11RNF1-KX90 | 5478347 | 606.25 | 0.978 | 0.944 | 0.567 | 91 | 93 | 93 | 1.282 |
| KA221S0587-XXXXH10XNF1-KX90 | 4491709 | 500.14 | 0.975 | 0.933 | 0.567 | 73.5 | 95 | 95 | 1.337 |
| KA221S0588-XXXXH10XNF1-KX90 | 5664198 | 664.85 | 0.970 | 0.924 | 0.569 | 97.4 | 93 | 93 | 1.174 |
| KA221S0589-XXXXH10XNF1-KX90 | 5040871 | 552.48 | 0.974 | 0.932 | 0.587 | 63.7 | 93 | 92 | 1.203 |
| KA221S0590-XXXXH10XNF1-KX90 | 4319411 | 494.48 | 0.976 | 0.934 | 0.572 | 78.2 | 92 | 91 | 1.282 |
| KA221S0591-XXXXH10XNF1-KX90 | 5234657 | 618.51 | 0.973 | 0.928 | 0.531 | 74 | 95 | 95 | 1.276 |
| KA221S0592-XXXXH10XNF1-KX90 | 6323763 | 739.89 | 0.973 | 0.929 | 0.569 | 116 | 92 | 92 | 1.234 |
| KA221S0593-XXXXH10XNF1-KX90 | 5765667 | 656.7 | 0.975 | 0.934 | 0.571 | 87.2 | 96 | 95 | 1.228 |
| KA221S0594-XXXXH10XNF1-KX90 | 6765880 | 783.02 | 0.971 | 0.925 | 0.566 | 117.9 | 95 | 95 | 1.215 |
| KA22290284-XXXXH14XNF1-KX90 | 3786391 | 417.18 | 0.977 | 0.938 | 0.567 | 62 | 94 | 93 | 1.291 |
| KA22290285-XXXXH14XNF1-KX90 | 4188025 | 470.1 | 0.977 | 0.938 | 0.569 | 72.6 | 93 | 92 | 1.252 |
| KA22290287-XXXXH14XNF1-KX90 | 4390584 | 519.48 | 0.978 | 0.939 | 0.565 | 78.1 | 95 | 95 | 1.26 |
| KA22290289-XXXXH14XNF1-KX90 | 3720380 | 422.82 | 0.984 | 0.952 | 0.573 | 67.5 | 88 | 87 | 1.298 |
| KA22290290-XXXXH14XNF1-KX90 | 4028297 | 454.96 | 0.977 | 0.938 | 0.571 | 68.6 | 86 | 85 | 1.248 |
| KA225P0357-XXXXH11RNF1-KX90 | 3850249 | 475.17 | 0.975 | 0.934 | 0.561 | 70.5 | 94 | 92 | 1.439 |
| KA225P0364-XXXXH11RNF1-KX90 | 2868379 | 368.54 | 0.976 | 0.936 | 0.562 | 54.6 | 94 | 89 | 1.436 |
| KA225P0365-XXXXH11RNF1-KX90 | 3646716 | 461.13 | 0.971 | 0.924 | 0.566 | 71.2 | 93 | 92 | 1.25 |

| **Table S5. Comparing the resistant mechanisms identified by CMT and WGS** | | |
| --- | --- | --- |
| Only CMT (n=15) | Only WGS (n=17) | CMT_WGS shared (n=25) |
| Trimethoprim/Sulfamethoxazole | Gentamicin A | Tobramycin |
| Tigecycline | Streptomycin | Piperacillin |
| Ceftazidime/Avibactam | Ceftiofur | Ceftazidime |
| Roxithromycin | Amoxicillin | Cefepime |
| Cefotetan | Ofloxacin | Cefazolin |
| Cefpodoxime | Ticarcillin/Clavulanic acid | Cefotaxime |
| Minocycline | Ampicillin/Clavulnic acid | Ceftriaxone |
| Ceftizoxime | Sulfisoxazole | Ticarcillin |
| Doripenem | Sulfamethoxazole tablets | Ampicillin |
| Nalidixic acid | Ceftaroline | Levofloxacin |
| Cefoperazone/Sulbactam | Penicillin G | Ciprofloxacin |
| Polymyxin | Trimethoprim | Moxifloxacin |
| ESBL detection | Cefixime | Norfloxacin |
| Gentamicin | Erythromycin | Imipenem |
| Nitrofurantoin | Florfenicol | Ertapenem |
|  | Chloramphenicol | Piperacillin/Tazobactam |
|  | Azithromycin | Cefoxitin |
|  |  | Aztreonam |
|  |  | Meropenem |
|  |  | Amoxicillin/Clavulanic acid |
|  |  | Cefuroxime |
|  |  | Cephalothin |
|  |  | Amikacin |
|  |  | Tetracycline |
|  |  | Doxycycline |
| CMT, conventional microbiological test; WGS, whole-genome sequencing | | |

| **Table S6. Multilocus sequencing typing (MLST) of MDR-Kp strains (N=18)** | |
| --- | --- |
| Type | N (%) |
| ST11 | 15 (83.3) |
| ST15 | 1 (5.6) |
| ST48 | 1 (5.6) |
| ST353 | 1 (5.6) |
